# Poor disease knowledge is associated with higher healthcare service use and costs among patients with cirrhosis: an exploratory study

**DOI:** 10.1186/s12876-022-02407-6

**Published:** 2022-07-14

**Authors:** Patricia C. Valery, Christina M. Bernardes, Kelly L. Hayward, Gunter Hartel, Katelin Haynes, Louisa G. Gordon, Katherine A. Stuart, Penny L. Wright, Amy Johnson, Elizabeth E. Powell

**Affiliations:** 1 QIMR Berghofer Medical Research Institute, 300 Herston Road, Herston, QLD 4006 Australia; 2 Centre for Liver Disease Research, Faculty of Medicine, The University of Queensland, Brisbane, QLD Australia; 3 Department of Gastroenterology and Hepatology, Princess Alexandra Hospital, Woolloongabba, QLD Australia; 4 Hepatitis Queensland, Coorparoo, QLD Australia

**Keywords:** Chronic liver disease, Patient knowledge, Hospital admissions, Healthcare cost, Survival

## Abstract

**Background:**

Optimal management of cirrhosis is complex, and patients often lack knowledge and skills, which can affect self-management. We assessed patient knowledge about cirrhosis and examined whether knowledge was associated with clinical outcomes, healthcare service use, and healthcare costs. A cross-sectional ‘knowledge survey’ was conducted during 2018–2020. We assessed patient knowledge about cirrhosis and explore whether knowledge was associated with clinical outcomes, healthcare service use, and costs.

**Methods:**

Patients with cirrhosis (n = 123) completed a ‘knowledge survey’. We calculated the proportion of correct answers to eight questions deemed to be “key knowledge” about cirrhosis by an expert panel, and dichotomized patients as ‘good knowledge’/‘poor knowledge’. Clinical data, healthcare costs, and health-related quality of life (SF-36) were available.

**Results:**

58.5% of patients had ‘good knowledge’ about cirrhosis. Higher education level was associated with higher odds of having ‘good knowledge’ about cirrhosis (adjusted-OR = 5.55, 95%CI 2.40–12.84). Compared to patients with ‘poor knowledge’, those with ‘good knowledge’ had a higher health status in the SF-36 physical functioning domain (*p* = 0.011), fewer cirrhosis-related admissions (adjusted incidence rate ratio [IRR] = 0.59, 95%CI 0.35–0.99) and emergency presentations (adj-IRR = 0.34, 95%CI 0.16–0.72), and more planned 1-day cirrhosis admissions (adj-IRR = 3.96, 95%CI 1.46–10.74). The total cost of cirrhosis admissions was lower for patients with ‘good knowledge’ (adj-IRR = 0.30, 95%CI 0.29–0.30).

**Conclusion:**

Poor disease knowledge is associated with increased use and total cost of healthcare services. Targeted educational interventions to improve patient knowledge may be an effective strategy to promote a more cost-effective use of healthcare services.

**Supplementary Information:**

The online version contains supplementary material available at 10.1186/s12876-022-02407-6.

## Background

Cirrhosis is the leading cause of liver-related death globally [1] and is an increasing contribution to the health burden of many countries [2]. In Australia, liver disease is among the top ten leading causes of years of life lost, and the disease burden increased during 2003–2015 [3]. In the past two decades, preventive and treatment strategies have been established to reduce transmission of viral hepatitis C and B [4, 5]. However, this effect is postulated to be offset by sustained hazardous alcohol consumption [6] and rising burden of non-alcoholic fatty liver disease (NAFLD) [7]. In fact, in the state of Queensland, the number of hospital admissions for cirrhosis increased 1.6-fold during 2008–2016 [8]. Although Australian data are limited [9], the productivity impacts and healthcare costs of managing advanced liver disease are staggering [10], and the disease has a substantial impact on patients’ activities of daily living and health-related quality of life [11].

Optimal management of cirrhosis can be challenging, with many patients required to follow complex medication regimens, dietary restrictions, and engage in disease monitoring activities. Chronic disease management is more effective if patients have the knowledge to manage their health [12]. However, previous studies have shown that patient knowledge about cirrhosis and self-care tasks is variable [13–16]. While most patients with cirrhosis understand the need to cease alcohol consumption, many lack understanding about key aspects of the management of cirrhosis complications, such as sodium restriction to control ascites and avoidance of constipation to prevent hepatic encephalopathy. In other chronic diseases, lack of knowledge and skills have been associated with poor self-management and lower levels of adherence to clinician recommendations [12]. People with low health literacy have a poorer comprehension of their disease [17, 18], poorer ability to take medications and interpret labels and health messages correctly without guidance [19], and these patients also have more hospitalisations [20, 21]. Moreover, experiences in other chronic disease settings (e.g. heart failure, chronic obstructive pulmonary disease, diabetes) with nurse-led patient education interventions demonstrate improvements in health-related quality of life, reduced hospital admissions and readmissions [22–24], and cost-effectiveness [22].

Aimed at informing the development and evaluation of an educational intervention specific to the needs of Australians with chronic liver disease, we assessed patient knowledge about cirrhosis and self-care tasks through a cross-sectional survey of a well-characterised cohort of patients with cirrhosis for whom detailed clinical data including hospital admissions, emergency department presentations, and costs were available. We also explored factors associated with patient knowledge and compared clinical outcomes (health-related quality of life and survival), healthcare service use (hospital admissions, emergency department presentations) and healthcare costs according to patient knowledge.

## Methods

### Study sample

**Fig. 1:**
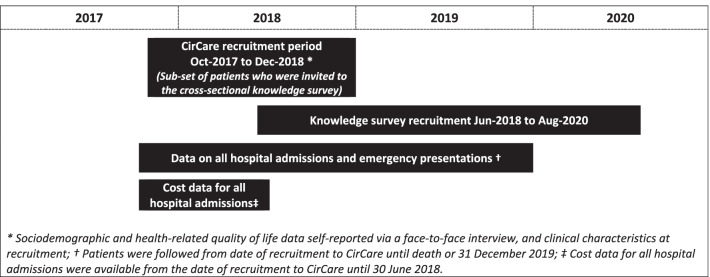
Timeline for recruitment and collection of outcome measures

Between Jun-2018 and Aug-2020, a cross-sectional knowledge study was conducted in a subset of patients enrolled in the CirCare study (Fig. 1). Briefly, CirCare is a prospective observational multicentre longitudinal study of 581 patients with cirrhosis who were consecutively recruited when they attended liver clinics or were admitted to one of five hospitals in Brisbane and Logan, Queensland, Australia between Jul-2016 and Dec-2018 [11]. Most patients (n = 568) included in the CirCare study had a one-off contact with the study nurse when they participated in the face-to-face interview to collect study data. Thirteen Indigenous patients were re-contacted to take part in a qualitative sub-study. No intervention, including education, was provided to patients through the CirCare study. Patients received routine review following the current model of care in the Hepatology clinic.

Patients with cirrhosis were eligible for the ‘knowledge survey’ if they had agreed to be recontacted for future studies, were not known to be deceased at the time the survey was posted, and if their last contact with researchers was after 1 October 2017 (to ensure relatively recent recruitment). A total of 292 surveys were mailed to eligible patients with instructions to return the completed anonymised survey using a prepaid envelope. A reminder was sent out by mail and, for those patients with telephone details available, a follow-up telephone call was attempted. Aboriginal and Torres Strait Islander patients (also referred to as Indigenous Australians) were provided with a second opportunity to participate in the ‘knowledge survey’ while taking part in a qualitative sub-study during April–July, 2020. Patient outcomes from the date of CirCare recruitment to Dec-2019 were obtained via data linkage. As data regarding date and cause of death were provided in Jun-2020, this information was not available when the knowledge survey was distributed.

### Study measurements

#### Knowledge survey

**Fig. 2:**
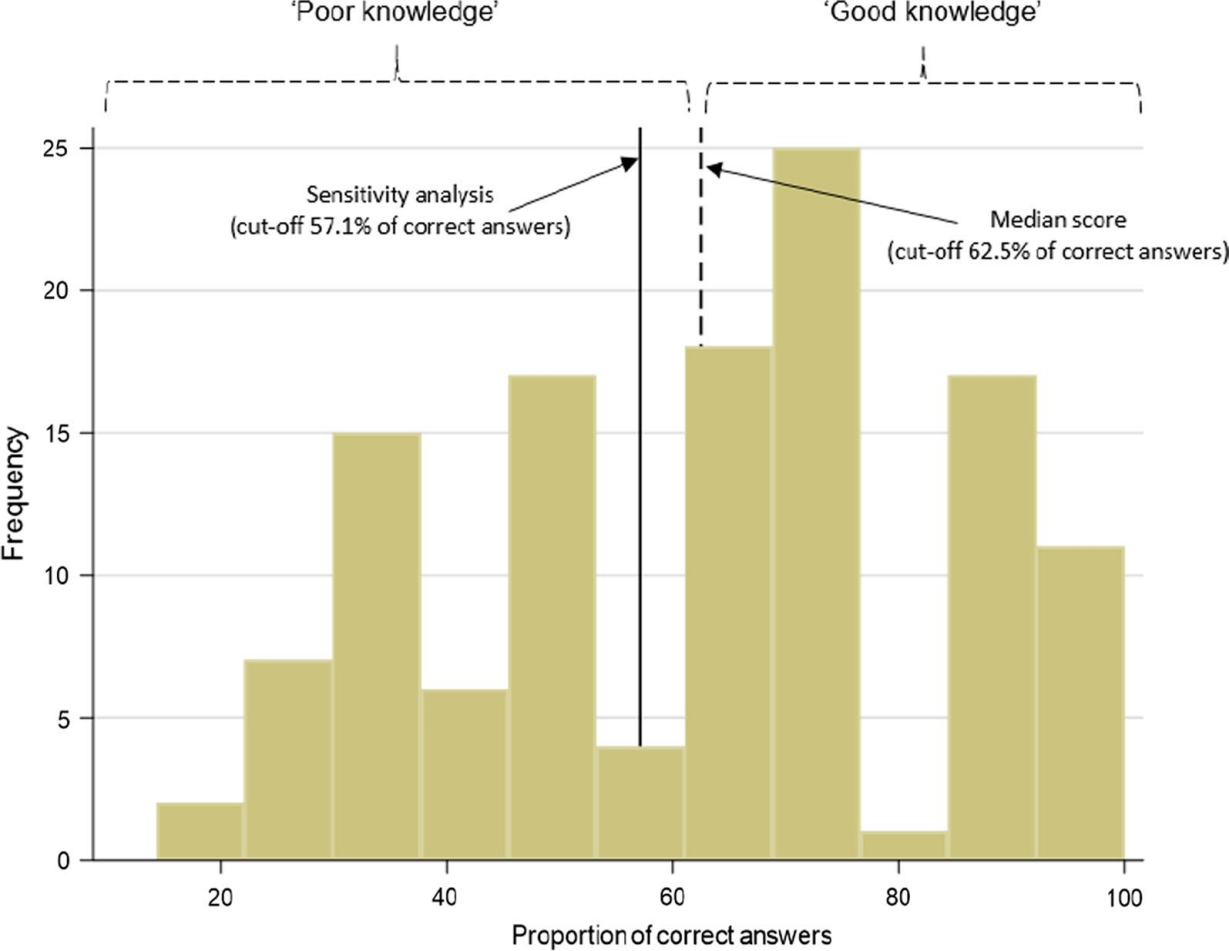
Frequency of participants and proportion of correct answers to the 8-item ‘knowledge survey’

The ‘knowledge survey’ (see Additional file 1: Table S1) included 17 questions derived from prior studies [13, 14]. An expert panel including two hepatologists, a liver nurse, and a liver pharmacist (EEP, KS, PW, and KH) subsequently reviewed the questions (blinded to the data) and unanimously deemed eight to be “key knowledge” about liver disease. Patients’ knowledge scores were calculated by assigning correct responses a score of 1, and incorrect or ‘I don’t know’ responses a score of 0. The proportion of correct answers were calculated for each patient and referred to here as the “key knowledge” score. The total score, over a range of 0–100%, was dichotomised using the median score as the cut-off point. Patients were categorised as ‘good knowledge’ for scores ≥ 62.5% (that is 5 out of 8 correct answers; Fig. 2), and ‘poor knowledge’ if < 62.5%.

#### Health related quality of life

Health-related quality of life data were self-reported via a face-to-face interview at recruitment into the CirCare study. The Short Form 36 (SF-36) [25], a widely used and validated quality of life tool, includes 36 questions grouped into 8 domains (general health, physical functioning, social functioning, bodily pain, role limitations due to physical problems (role physical), emotional wellbeing, role limitations due to emotional problems (role emotional) and vitality). Health-related quality of life raw domain scores were transformed to range from 0 to 100, with a higher score indicating a higher health status.

#### Outcome data

Health service use data were obtained via data linkage from the Queensland Hospital Admitted Patient Data Collection database and the Emergency Data Collection database that contain information on all hospital episodes of care for patients admitted to Queensland public and private hospitals. Hospital admissions were categorised as ‘cirrhosis admissions’ based on recorded ICD‐10‐AM codes as previously described [26]. The accuracy of this algorithm for identification of patients with cirrhosis has been reported to have an 88% positive predictive and 76% negative predictive value [26]. Emergency presentations were categorised as ‘cirrhosis-related presentations’ if they had a primary or other diagnosis of cirrhosis [26], cirrhosis-related diagnosis, or cirrhosis-related complications; namely chronic hepatic failure, portal hypertension, hepatorenal syndrome, spontaneous bacterial peritonitis, ascites, variceal bleeding, hepatic encephalopathy, jaundice, or alcohol related presentation (e.g. alcoholic hepatitis, alcoholic liver disease unspecified). Death Registration Data and Cause of Death Unit Record File were obtained from the Australian Bureau of Statistics. The National Hospital Cost Data Collection database was the primary source of cost data for all hospital admissions at public and private hospitals. Costs included aggregated direct plus overhead costs. Hospital admissions, emergency presentations and death data were available from the CirCare study recruitment date to Dec-2019. Cost data were available from the CirCare study recruitment date to Jun-2018. A diagram showing the timeline for recruitment and collection of outcome measures is displayed in Fig. 1.

#### Sociodemographic and clinical characteristics

Sociodemographic data were self-reported at recruitment into the CirCare study. Place of residence was categorised according to rurality of residence [27] and the Index of Relative Socioeconomic Advantage and Disadvantage [28]. Clinical information at the time of recruitment was extracted from patients’ medical records. Severity of disease was classified using the Child–Pugh class and by absence (compensated cirrhosis) versus presence of cirrhosis complications (e.g. ascites, hepatic encephalopathy). Comorbidity burden was measured using the Charlson Comorbidity Index [29] using validated coding algorithms [30].

### Data analysis

Data analyses were conducted using Stata/SE (version 15; Stata Corporation, College Station, TX). Descriptive analyses of patient characteristics and responses to the knowledge survey were presented as frequency (percentages) and mean (standard deviation, SD).

Multivariable logistic regression analysis reported odds ratios (ORs) with associated 95% confidence intervals (CIs) to examine factors that were independently associated with “key knowledge”. The decision as to which independent variables were included was first determined based on the results of bivariable analyses. We then ran multivariable analysis to appreciate the extent of confounding and applied stepwise model selection (*p* = 0.20 as the significance level at which variables were entered to or removed from the model). As longer duration of disease may provide patients with more opportunities to receive information about cirrhosis, we have adjusted the estimates for duration of disease. The final model for “key knowledge” included the following covariates: presence of complications of cirrhosis, age, education level, and duration of cirrhosis.

The rate of hospital admissions and emergency department presentations was calculated using person days at risk (PDAR) as a denominator. Cases were followed from date of recruitment date to CirCare until death or December 31, 2019, whichever came sooner. Poisson regression was used to compare rate of admission or emergency department presentation according to “key knowledge” status (incidence rate ratios (IRR) and 95%CIs were reported). Education level, socioeconomic status, duration of cirrhosis and severity of liver disease (measured using presence of cirrhosis complications) were included in the model. As Child–Pugh score was unavailable for 3 patients, the presence of cirrhosis complications was included in multivariable analysis as a marker of severity of liver disease.

We reported IRRs to describe the ratio of costs of hospital admissions according to “key knowledge” status. PDAR included data from the CirCare study recruitment date until the date of death or June 30, 2018, whichever came sooner. As comorbidity burden and severity of cirrhosis are associated with health service use, we have included Charlson Comorbidity Index and presence of complications of cirrhosis in the model, along with education level [29, 31].

Sensitivity analyses were carried out by: (1) using a cut-off of 57.1% on the 8-item score (58.5% of study participants had ≥ 51.7% of correct answers; Fig. 2); and (2) by including three extra items deemed to be “key knowledge” of liver disease by half of the experts and using a cut-off of ≥ 60.0% (median score of the 11-item survey).

Cumulative overall survival estimates according to patient knowledge were calculated using the Kaplan–Meier method (log-rank statistic). All cases were followed from the date of completion of the knowledge survey until date of death or December 31, 2019, whichever came sooner. Multivariable Cox regression analysis reported in terms of hazard ratios (HRs) with associated 95%CIs was used to assess the differences in survival according to “key knowledge” status. The vce(robust) option was used to obtain robust standard errors for the parameter estimates to control for mild violations of underlying assumptions. All *p* values were 2-sided.

## Results

Of the 292 patients invited to respond to the knowledge survey, 15 had died, 123 returned the survey (response rate 44.4%), 2 patients declined via response letter, and 152 did not return the survey. The sociodemographic and clinical characteristics of the invited patients who did not complete/return the knowledge survey (n = 169) were comparable to survey responders (all *p* > 0.05; see Additional file 1: Table S2) with the exception of a higher proportion of Indigenous patients among ‘knowledge survey’ respondents (10.6% vs. 3.0%; *p* = 0.012), reflecting the extra effort in recruiting this subgroup of patients. Six additional Indigenous Australians answered the knowledge survey through the ‘second opportunity’ pathway, resulting in a response rate of 72% for Indigenous Australians versus 43% for non-Indigenous Australians (*p* = 0.014). Without this second opportunity to participate in the survey (excluding the abovementioned six Indigenous Australians), the response rates were 39% versus 43%, respectively (*p* = 0.77). The sociodemographic and clinical characteristics of six Indigenous patients recruited through the ‘second opportunity’ pathway were comparable to Indigenous patients (n = 7) and to all patients (n = 117) included in the mainstream recruitment (all *p* > 0.05; see Additional file 1: Table S3). Data presented hereafter describe the 123 patients included in the current study.

Most patients (n = 104, 84.6%) were recruited from outpatient clinics at the hospitals included in the study, 15.4% (n = 19) were inpatients at recruitment. The median time from CirCare recruitment to completion of the knowledge survey was 126 days (interquartile range 68–224 days). At the time of recruitment to CirCare, patients’ mean age was 60.7 ± 10.8 years, 65.9% were male, 72.4% were Australian born, 10.6% identified themselves as Indigenous Australians, 47.2% had formal education to Junior High School level or less, 43.9% lived in most disadvantaged areas (bottom two quintiles of socioeconomic status), and 81.3% lived in a major city area, which is reflective of the study recruitment sites.

An approximate date of cirrhosis diagnosis was available for 85 (69.1%) patients. 28 patients (22.8%) out of 123 were diagnosed three or more years prior to CirCare recruitment, 32 (26.0%) were diagnosed 2–3 years prior, and 25 (20.3%) were diagnosed within the previous year. Alcohol-related cirrhosis was the primary liver disease aetiology for 35.8% of patients, followed by NAFLD in 30.1%, and hepatitis C virus in 26.0%. Alcohol was a cofactor for 65.9% of patients and NAFLD was a cofactor for 50.4%. Over two-thirds of the patients had Child–Pugh A cirrhosis (68.3%), and 29.3% had at least one cirrhosis complication (decompensated disease) documented in their medical notes at recruitment. Nearly two-thirds of the patients (61.8%) had a least one comorbidity; the most common comorbidity listed in the Charlson Comorbidity Index was diabetes, which was present in 39.8% of patients.

### Patient knowledge about liver disease

Patients’ key knowledge of cirrhosis and self-care tasks was variable (Table 1). Most patients correctly reported that they had cirrhosis (87.5%), the aetiology of their liver disease (e.g. hepatitis B, alcohol, fatty liver; 85.2%), and awareness of the need to cease alcohol intake (93.5%). Fewer than half of the patients responded correctly to questions about safe over-the-counter medications for pain relief (40.6%), use of sleeping tablets and calmatives (49.6%), “natural” remedies, herbs, or supplements (48.0%), and preventive healthcare activities such as ultrasound screening for liver cancer (33.9%). Only 38.1% of patients correctly answered whether they had compensated or decompensated cirrhosis. See Additional file 1: Table S1 for the responses to all items of the knowledge survey.

**Table 1:** Proportion of correct responses to “key knowledge” questions

“Key knowledge” questions	N = 123 (%)
1. Do you have cirrhosis (scarring of the liver)?*	98 (87.5%)
2. What type of liver disease do you have?**	104 (85.2%)
3. People who have cirrhosis should not drink alcohol	115 (93.5%)
4. It is safe for people with cirrhosis to take sleeping tablets and calmatives without discussing it with their liver doctor	61 (49.6%)
5. It is safe for people with cirrhosis to take natural remedies, herbs, or supplements without discussing it with their liver doctor	59 (48.0%)
6. For people with cirrhosis who have minor aches or pains, the following over-the-counter medications are safe to take:^¥^ (1) naproxen; (2) paracetamol; (3) ibuprofen; (4) aspirin; (5) diclofenac	39 (40.6%)
7. When should people with cirrhosis be screened for liver cancer?^₤^ (1) Never, people with cirrhosis are not at increased risk for liver cancer; (2) People with cirrhosis should be screened for liver cancer when their cirrhosis is decompensated; (3) People with cirrhosis should have an ultrasound every 6 months to screen for liver cancer; (4) I don’t know	75 (62.0%)
8. People with cirrhosis have an ultrasound every 6 months in order to:^₤^ (1) Determine liver function; (2) Look for gallstones; (3) Look for liver cancer; (4) I don’t know	41 (33.9%)

*Data was missing for 11 patients

**Data was missing for 1 patient

^¥^Examples of commonly used commercial names of medications were included in the survey provided to patients; 21 patients noted that they did not take the listed over-the-counter medications and data was missing for 6 patients

^₤^Data was missing for 2 patients

Based on the eight items identified as “key knowledge” by an expert panel, 58.5% of the patients had ‘good knowledge’ about cirrhosis and 41.5% had ‘poor knowledge’. In bivariable analysis, older age (≥ 65 years) was associated with ‘poor knowledge’ (OR = 0.42, 95%CI 0.20–0.89), and higher education level (Senior High School or more) was associated with ‘good knowledge’ (OR = 5.45, 95%CI 2.49–11.98; Table 2). In multivariable analysis, the only factor significantly associated with patient knowledge was education level. Following adjustment for age, socioeconomic status, presence of cirrhosis complications, and duration of cirrhosis, having a higher level of education was associated with over fivefold odds of having ‘good knowledge’ about liver disease (adj-OR = 5.55, 95%CI 2.40–12.84).

**Table 2:** Factors associated with “key knowledge” about liver disease

	Poor knowledge N = 51 (%)	Good knowledge N = 72 (%)	OR (95%CI)	Adjusted-OR (95%CI)*
*Age group*				
18–64 years	25 (49%)	50 (69%)	1.00	1.00
≥ 65 years	26 (51%)	22 (31%)	0.42 (0.20–0.89)	0.46 (0.19–1.14)
*Gender*				
Female	20 (39%)	22 (31%)	1.00	1.00
Male	31 (61%)	50 (69%)	1.47 (0.69–3.12)	1.46 (0.57–3.77)
*Indigenous status*				
Non-indigenous	44 (86%)	66 (92%)	1.00	1.00
Indigenous	7 (14%)	6 (8%)	0.57 (0.18–1.82)	0.78 (0.25–2.45)
*Education*				
Junior high school or less	36 (71%)	22 (31%)	1.00	1.00
Senior high school or more (e.g. trade qualification, university degree)	15 (29%)	50 (69%)	5.45 (2.48–11.98)	5.55 (2.40–12.84)
*Socioeconomic status*				
Q1 most affluent/Q2	16 (31%)	37 (51%)	1.00‡	1.00^‡^
Q3	10 (20%)	6 (8%)	0.26 (0.08–0.84)	0.24 (0.06–0.91)
Q4/ Q5 most disadvantage	25 (49%)	29 (40%)	0.50 (0.22–1.11)	0.47 (0.19–1.12)
*Presence of complications of cirrhosis*				
Compensated	39 (76%)	48 (67%)	1.00	1.00
Decompensated	12 (24%)	24 (33%)	1.63 (0.72–3.67)	1.85 (0.77–4.43)
*Duration of cirrhosis relative to recruitment in the CirCare study*				
Diagnosed < 1 year prior	11 (22%)	14 (19%)	1.00‡	1.00^‡^
Diagnosed 2–3 years prior	14 (27%)	18 (25%)	1.01 (0.35–2.91)	1.46 (0.40–5.36)
Diagnosed > 3 years prior	10 (20%)	18 (25%)	1.41 (0.47–4.29)	2.17 (0.56–8.40)
Unknown	16 (31%)	22 (31%)	1.08 (0.39–3.01)	1.06 (0.33–3.38)

Data presented as odds ratios (OR) and 95% confidence intervals (CI). The vce(robust) option was used to obtain robust standard errors for the parameter estimates to control for mild violations of underlying assumptions

*Multivariable logistic regression model included education level, socioeconomic status, age, presence of complications of cirrhosis and duration of cirrhosis

^‡^*p* value > 0.05

### Patient outcomes

#### Quality of life

**Fig. 3:**
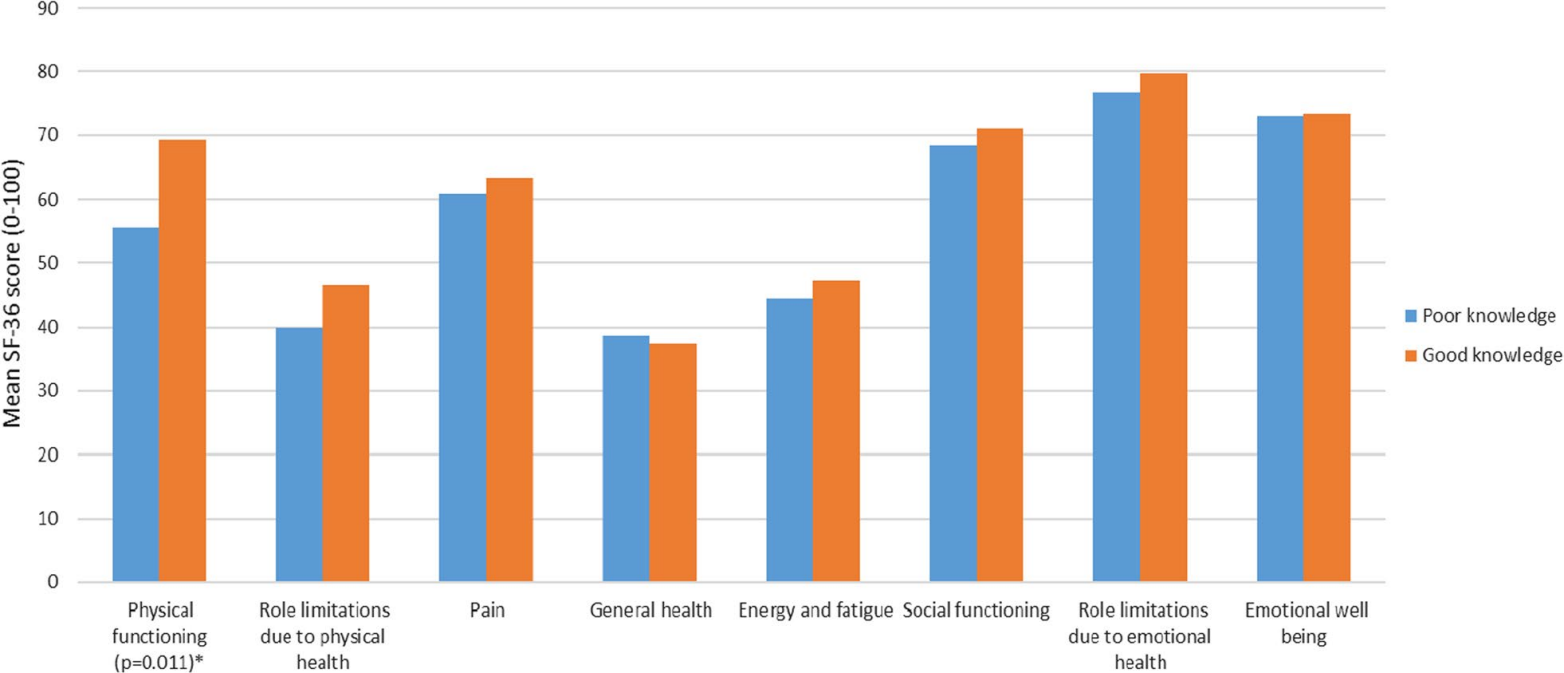
Mean SF-36 scores according to patient knowledge. *Note*: Missing SF-36 scores for 2 patients; *Multivariable logistic regression adjusted for education level, socioeconomic status, presence of complications of cirrhosis, and duration of cirrhosis

In a multivariable analysis adjusted for education level, socioeconomic status, presence of cirrhosis complications and duration of cirrhosis, patients with ‘good knowledge’ had a significantly higher score in the SF-36 domain related to physical functioning (*p* = 0.011; with a higher score indicating a higher health status) compared to those with ‘poor knowledge’. There were no significant differences in the other SF-36 domains of health-related quality of life (Fig. 3 and Additional file 1: Table S4).

#### Health service use and costs

Over one-third of the 121 patients included in analysis of health service use had at least one admission for cirrhosis after recruitment into the CirCare study (35.0% of patients with ‘poor knowledge’ and 36.0% with ‘good knowledge’). In multivariable analysis adjusted for education level, socioeconomic status, presence of cirrhosis complications and duration of cirrhosis, patients with ‘good knowledge’ about their liver disease had significantly fewer all-cause hospital admissions, admissions via the emergency department, and emergency presentations (Table 3). In particular, patients with good knowledge had 76% fewer all-cause admissions (adjusted IRR = 0.24, 95%CI 0.20–0.29; *p* < 0.001), 41% fewer admissions for cirrhosis (adj-IRR = 0.59, 95%CI 0.35–0.99; *p* = 0.046), 66% fewer cirrhosis-related emergency presentations (adj-IRR = 0.34, 95%CI 0.16–0.72; *p* = 0.005) and more planned 1-day cirrhosis admissions, reflected by an incidence rate ratio of 3.96 (95%CI 1.46–10.74; *p* = 0.007).

**Table 3:** Adjusted incidence rate ratios and cost ratios according to knowledge score among 121 patients

Data source: Queensland hospital admitted patient data collection	IRR (95%CI)*	*p* value	Adjusted-IRR (95%CI)*	*p* value
All-cause admission	0.42 (0.35–0.49)	< 0.001	0.24 (0.20–0.29)	**< 0.001**
Cirrhosis admission	1.21 (0.82–1.79)	0.338	0.59 (0.35–0.99)	**0.046**
Planned one-day admission (cirrhosis admission)	4.59 (1.79–11.73)	**0.001**	3.96 (1.46–10.74)	**0.007**
Admitted via the emergency department (any admission)	0.70 (0.51–0.97)	**0.034**	0.51 (0.35–0.76)	**0.001**
Admitted via the emergency department (cirrhosis admission)	0.90 (0.57–1.41)	0.646	0.57 (0.32–1.00)	0.050

Data source: emergency data collection	IRR (95%CI)*	*p* value	IRR (95%CI)*	*p* value
Emergency presentation (any reason)	0.76 (0.58–1.00)	**0.048**	0.62 (0.44–0.85)	**0.004**
Cirrhosis-related emergency presentation	0.70 (0.40–1.25)	0.229	0.34 (0.16–0.72)	**0.005**

Data source: National hospital cost data collection	Cost ratio (95%CI)**	*p* value	Cost ratio (95%CI)**	*p* value
Total cost for any admission	0.25 (0.24–0.25)	**< 0.001**	0.09 (0.08–0.09)	**< 0.001**
Total cost for cirrhosis admissions	0.54 (0.53–0.54)	**< 0.001**	0.30 (0.29–0.30)	**< 0.001**

Bold values indicates statistically significant (*p* < 0.05)

Incidence rate ratio (IRR) and cost ratio with poor knowledge as reference group

Two patients for whom we did not have hospital admission data were excluded from these analyses

*Multivariable Poisson regression model included education level, socioeconomic status, presence of complications of cirrhosis and duration of cirrhosis

**Multivariable Poisson regression model included education level, Charlson Comorbidity Index, and presence of complications of cirrhosis

There was also a notable discrepancy in the cost for hospital admissions according to patient knowledge. Compared to those with ‘poor knowledge’, the total cost of cirrhosis admissions was 70% lower for patients with ‘good knowledge’ (adj-IRR = 0.30, 95%CI 0.29–0.30; *p* < 0.001) during the follow-up period.

#### Sensitivity analyses

When the analyses were repeated using (1) a “key knowledge” score cut-off of 57.1% on the 8-item score and (2) the 11-item score (≥ 60.0% cut-off), the results were similar to the main analysis. Of note was a stronger association with planned 1-day cirrhosis admissions using the 11-item score (IRR = 10.9, 95%CI 4.17–28.55; *p* < 0.001). See Additional file 1: Table S5 for the results of sensitivity analyses.

#### Survival

At the end of the follow up period 12.2% of patients were deceased (11.1% of patients with ‘good knowledge’ vs. 13.7% of patients with ‘poor knowledge’), with a median time from the completion of the knowledge survey to date to death of 1.14 years (IQR 0.94–1.33) and 0.95 years (IQR 0.81–1.17), respectively. The 1-year survival for patients with ‘good knowledge’ was 92.9% (95%CI 81.5–96.5) vs 89.2% (95%CI 75.9–95.4) for patients with ‘poor knowledge’. These similarities were reflected in the hazard ratio of 0.54 (95%CI 0.17–1.65; *p* = 0.279; adjusted for education level, socioeconomic status, presence of complications of cirrhosis and duration of cirrhosis, with ‘poor knowledge’ as the reference group.

## Discussion

The main finding from this study was that poor knowledge among patients with cirrhosis was associated with higher healthcare service utilization and expenditures. Patients with less awareness about key aspects of their liver disease and self-care tasks incurred more hospital admissions and emergency presentations compared to those with good knowledge.

In Australia, the management of patients with cirrhosis is largely guided by liver specialists with the goal of slowing the progression of liver disease, treating symptoms and complications of cirrhosis, including the early detection and management of primary liver cancer and gastroesophageal varices. A high level of patient engagement in cirrhosis care is paramount. However, this study found that greater than half of the participants lacked specific knowledge about their disease, and that ‘poor knowledge’ was associated with higher rates of hospital admissions and emergency department presentations independently of liver disease severity. Conversely, those with ‘good knowledge’ had higher rates of planned one-day cirrhosis admissions which usually represent delivery of planned endoscopic or therapeutic procedures, and suggests a higher level of engagement in preventative healthcare measures among these patients.

Low health literacy among patients with cirrhosis has been previously reported [13, 14, 32], and delivery of liver health education has been shown to improve patient knowledge about cirrhosis [14, 15, 32, 33]. Educational intervention as simple as a one-page letter with basic information about the risk of liver cancer in patients with cirrhosis and recommendation to undergo liver cancer surveillance can increase the rate of surveillance compared with usual care [34]. In a review of interventional studies [35] examining the effectiveness of self-management programmes for 299 patients with cirrhosis, while the content of interventions varied substantially across the four identified studies, patient education was a common feature. One randomised‐controlled trial (RCT) included in this review which included health service use as an endpoint reported that the rate of attendance for planned outpatient care (secondary end point) was higher in the intervention group compared to usual care, but no difference was seen in liver-related occupied bed days (primary end point) [36]. In Australia, Hayward et al.’s RCT showed that multifaceted pharmacist-led medication and disease education for patients with decompensated cirrhosis improved not only patients’ knowledge about cirrhosis but also significantly reduced unplanned hospital admissions [37, 38].

There is a paucity of data about the association between health-related quality of life and health literacy. In the abovementioned review [35], two RCTs which included health-related quality of life as an endpoint reported no statistical difference between the intervention and control groups [36, 39]. In Hayward et al.’s study, patients who received the education intervention also experienced improved quality of life [38]. In this study, quality of life was assessed using a cirrhosis-specific quality of life tool [40] and improvement of scores from baseline to follow up were seen among patients who received the intervention, whereas usual care patients did not improve [38]. Zhang et al. showed that health education in a hospital setting in China improved patients’ understanding of key aspects of cirrhosis and how to manage it, and that led to improved health-related quality of life [33]. However in the latter study, it was not clear what measurement tool was used to assess quality of life [33]. In a US study of over 500 veterans, Rogal et al. showed that poor knowledge about cirrhosis symptoms was linked to reduced health-related quality of life [41]. In the US study, being “unsure about cirrhosis symptoms” was used as a proxy measure for disease knowledge as being uncertain about their disease severity or prior complications of cirrhosis could lead to patients not seeking timely action for ascites or a variceal bleed [41]. In our current study, patients with ‘poor knowledge’ about liver disease had a significantly lower quality of life related to physical functioning.

Chronic liver diseases, including cirrhosis and liver cancer disproportionately affect Indigenous Australians, and are important contributors to the mortality gap between Indigenous and non-Indigenous Australian adults [42]. Compared to non-Indigenous Queenslanders, the hospitalization rate for cirrhosis was 3.4 times higher for Indigenous Queenslanders [8], and survival was poorer during 2008–2016 [43]. Health literacy levels among Indigenous Australians are lower than among non-Indigenous Australians [19]. Understanding health literacy of Indigenous patients with cirrhosis is an important step in working toward improving health outcomes for this population [44]. While the extra opportunity to complete the knowledge survey offered to Indigenous patients may have introduced bias due to a differential response rate according to Indigenous status, given the exploratory nature of this study and the importance of including Indigenous Australians in any study of chronic liver disease, we believe the differential response rate was justified. Nevertheless, Indigenous status was not associated with “key knowledge” about liver disease in this study and the sociodemographic and clinical characteristics of Indigenous patients did not vary according to recruitment strategy.

### Limitations

Our study had limitations that must be considered when interpreting the results. Patients were recruited from liver specialist clinics, and those with cognitive impairment or inability to communicate were not included in the study. While the CirCare study included some patients from a non-English speaking background, many were excluded because an interpreter was not available to assist with the interview. The CirCare study was not an interventional study and patients recruited received usual care. Nevertheless, patients managed in liver specialist clinics and who access liver specialist nurses may be more likely to have better knowledge about cirrhosis than those managed by general physicians or general practitioners. Consequently, findings from this multicentre study may not be directly generalizable to all patients with cirrhosis in Australia. The 8-item ‘knowledge survey’ was not validated and was administered at a single time-point. Ramachandran et al. [45] developed and validated a 7-item survey about complications of cirrhosis to assess knowledge and self-management in patients with decompensated cirrhosis. The validated 7-item survey was developed after the data were collected in the CirCare study, and the 7-item questions are different from the 8-item survey used here. The former [45] focuses on recognition of “acute issues” that need hospital presentation, whereas our 8-item survey addresses knowledge that might prevent acute issues/decompensation events. Although the cost data had good hospital coverage and consistency, it did not allow for a detailed breakdown of the specific resources used, and only included direct hospital costs. Indirect expenses incurred by patients for stopping or reducing employment, travel, parking or over-the-counter medicines were not considered. Moreover, the use of primary health care services and the costs of these services were also not evaluated. These data could have provided further insights into resource allocation and should be considered in future prospective studies. Key data were gathered at recruitment (namely health-related quality of life, sociodemographic and clinical data), and the knowledge survey was conducted approximately 4 months later. As physical and emotional well-being may change within this time frame, health-related quality of life and clinical data at the time of completion of the knowledge survey may differ from data collected at recruitment. This may be particularly relevant for patients with decompensated cirrhosis who were recruited when admitted with complications of cirrhosis. Finally, due to the small sample size our findings should be interpreted with caution as there may have been differences that the study did not detect.

## Conclusions

Health literacy is a modifiable factor. Improving the knowledge of patients with chronic liver disease using targeted educational interventions may be an effective strategy to promote a more cost-effective use of healthcare services with fewer preventable emergency department visits and greater use of planned admissions. With the increasing burden of chronic liver disease globally [2], there is a need to develop appropriate chronic liver disease education for patients with cirrhosis. While there has not been comprehensive research to understand the association between health literacy and healthcare service use and costs and patient outcomes in cirrhosis, our study undertaken in a country with a universal health-care system showed that poor disease knowledge is associated with greater use of healthcare services. This may reflect poorer health status and quality of life, and less effective use of available health services.

## Supplementary Information

**Additional file 1**.** Table S1**. Proportion of correct responses to the knowledge survey questions.** Table S2**. Sociodemographic and clinical characteristics of respondents (n=123) and non-respondents (n=169) of the ‘knowledge survey’.** Table S3**. Sociodemographic and clinical characteristics of Indigenous Australians according to recruitment method: ‘second opportunity’ pathway (n=6) versus mainstream recruitment (all patients n=117 and Indigenous patients n=6).** Table S4**. Mean SF-36 scores according to patient knowledge.** Table S5**. Adjusted incidence rate ratios and cost ratios according to knowledge score: sensitivity analyses carried using a cut-off of 57.1% on the 8-item score; and using a cut-off of ≥60.0% on the 11-item survey among 121 patients ‡.

## Data Availability

The data that support the findings of this study contain potentially sensitive and/or identifying information that could compromise the privacy of the participants. Therefore data are not publicly available. Data may, however, be available from the authors upon reasonable request with approval from relevant ethics committees.

## Abbreviations

CI: Confidence interval
HR: Hazard ratio
IRR: Incidence rate ratio
NAFLD: Non-alcoholic fatty liver disease
OR: Odds ratio
PDAR: Person days at risk
*p*: *p* value
RCT: Randomised‐controlled trial
SF-36: Short form 36
SD: Standard deviation
