# Supplementary material for: Poor disease knowledge is associated with higher healthcare service use and costs among patients with cirrhosis: an exploratory study
Source: BMC Gastroenterol. 2022 Jul 14;22:340. doi: 10.1186/s12876-022-02407-6 (PMC9284723; doi:10.1186/s12876-022-02407-6)
Supplement: Supplementary file 1 — Additional file 1. Table S1. Proportion of correct responses to the knowledge survey questions. Table S2. Sociodemographic and clinical characteristics of respondents (n=123) and non-respondents (n=169) of the ‘knowledge survey’. Table S3. Sociodemographic and clinical characteristics of Indigenous Australians according to recruitment method: ‘second opportunity’ pathway (n=6) versus mainstream recruitment (all patients n=117 and Indigenous patients n=6). Table S4. Mean SF-36 scores according to patient knowledge. Table S5. Adjusted incidence rate ratios and cost ratios according to knowledge score: sensitivity analyses carried using a cut-off of 57.1% on the 8-item score; and using a cut-off of ≥60.0% on the 11-item survey among 121 patients ‡. [file 12876_2022_2407_MOESM1_ESM.docx]

**Additional file 1: Table S1**. Proportion of correct responses to the knowledge survey questions

|  | **Number who answered question**  **N** | **Correct answers**  **N (%)** |
| --- | --- | --- |
| **Questions about “your liver disease”** |  |  |
| 1. Do you have cirrhosis (scarring of the liver)? **†‡** | 112 | 98 (87.5%) |
| 1. What type of liver disease do you have? **†‡** | 122 | 104 (85.2%) |
| 1. What stage of cirrhosis do you have? **‡** | 105 | 40 (38.1%) |
| **True or false questions** |  |  |
| 1. People who have cirrhosis should not drink alcohol **†‡** | 123 | 115 (93.5%) |
| 1. People with cirrhosis should get the flu shot every year **‡** | 121 | 64 (52.9%) |
| 1. People with cirrhosis should be vaccinated against hepatitis A and hepatitis B **‡** | 121 | 46 (38.0%) |
| 1. It is safe for people with cirrhosis to take sleeping tablets and calmatives without discussing it with their liver doctor **†‡** | 123 | 61 (49.6%) |
| 1. It is safe for people with cirrhosis to take natural remedies, herbs, or supplements without discussing it with their liver doctor **†‡** | 123 | 59 (48.0%) |
| 1. It is not safe for people with cirrhosis to take natural remedies such as Comfrey, Chinese ginseng, Valerian or Echinacea | 121 | 32 (26.4%) |
| 1. It is safe for people with cirrhosis to take ‘Liver Detox’ supplements for liver health | 121 | 24 (19.8%) |
| 1. It is safe for people with fluid in the belly (ascites) to take Berocca | 123 | 22 (17.9%) |
| 1. Medications to lower cholesterol (“statins”) such as atorvastatin and rosuvastatin are safe for people with cirrhosis * | 123 | 23 (18.7%) |
| 1. People with cirrhosis always have abnormal liver blood tests | 119 | 27 (22.7%) |
| **Multiple choice questions** |  |  |
| 1. For people with cirrhosis who have minor aches or pains, the following over-the-counter medications are safe to take: * **†‡**   (i) naproxen; (ii) paracetamol; (iii) ibuprofen; (iv) aspirin; (v) diclofenac | 96 ₤ | 39 (40.6%) |
| 1. When should people with cirrhosis be screened for liver cancer? **†‡** (i) Never, people with cirrhosis are not at increased risk for liver cancer; (ii) People with cirrhosis should be screened for liver cancer when their cirrhosis is decompensated; (iii) People with cirrhosis should have an ultrasound every 6 months to screen for liver cancer; (iv) I don’t know | 121 | 75 (62.0%) |
| 1. People with cirrhosis get an endoscopy in order to: (i) Look for cancer; (ii) Look for varices (blood vessels that can bleed) in the oesophagus (food pipe) and stomach; (iii) Determine liver function; (iv) I don’t know | 120 | 51 (42.5%) |
| 1. People with cirrhosis have an ultrasound every 6 months in order to: **†** **‡** (i) Determine liver function; (ii) Look for gallstones; (iii) Look for liver cancer; (iv) I don’t know | 121 | 41 (33.9%) |

**†** These questions (n=8) were included in the overall assessment of “key knowledge” of liver disease; **‡** These questions (n=11) were included in the sensitivity analysis for the overall assessment of “key knowledge”; * These questions included examples of commonly used commercial names of medications; ₤ 21 patients noted that they did not take the listed over-the-counter medications and data was missing for 6 patients.

**Additional file 1: Table S2**. Sociodemographic and clinical characteristics of respondents (n=123) and non-respondents (n=169) of the ‘knowledge survey’

|  | |  | | | **Non-respondents** | **Respondents** |  |
| --- | --- | --- | --- | --- | --- | --- | --- |
|  | |  | | | N=169 | N=123 | **p-value** |
| **Gender** | Female | | | | 51 (30.2%) | 42 (34.1%) | 0.47 |
|  | Male | | | | 118 (69.8%) | 81 (65.9%) |  |
| **Country of birth** | Australia | | | | 126 (74.6%) | 89 (72.4%) | 0.67 |
|  | Overseas | | | | 43 (25.4%) | 34 (27.6%) |  |
| **Indigenous status** | Indigenous | | | | 5 (3.0%) | 13 (10.6%) | 0.012 |
|  | Non-Indigenous | | | | 164 (97.0%) | 110 (89.4%) |  |
| **Education** | Junior High/less | | | | 81 (47.9%) | 58 (47.2%) | 0.90 |
|  | Senior High/higher | | | | 88 (52.1%) | 65 (52.8%) |  |
| **Socioeconomic status** | Q1 most affluent | | | | 37 (21.9%) | 25 (20.3%) | 0.72 |
|  | Q2 | | | | 47 (27.8%) | 28 (22.8%) |  |
|  | Q3 | | | | 19 (11.2%) | 16 (13.0%) |  |
|  | Q4 | | | | 34 (20.1%) | 32 (26.0%) |  |
|  | Q5 Most disadvantage | | | | 32 (18.9%) | 22 (17.9%) |  |
| **Remoteness of residence** | Major city area | | | | 144 (85.2%) | 100 (81.3%) | 0.37 |
|  | Outside major city area | | | | 25 (14.8%) | 23 (18.7%) |  |
| **Primary liver disease aetiology** | Alcohol | | | | 55 (32.5%) | 44 (35.8%) | 0.14 |
|  | HCV | | | | 50 (29.6%) | 32 (26.0%) |  |
|  | NAFLD/NASH | | | | 37 (21.9%) | 37 (30.1%) |  |
|  | other | | | | 16 (9.5%) | 8 (6.5%) |  |
|  | HBV | | | | 11 (6.5%) | 2 (1.6%) |  |
| **Alcohol as cofactor** | no | | | | 64 (37.9%) | 42 (34.1%) | 0.51 |
|  | yes | | | | 105 (62.1%) | 81 (65.9%) |  |
| **NAFLD/NASH as cofactor** | no | | | | 85 (50.3%) | 61 (49.6%) | 0.91 |
|  | yes | | | | 84 (49.7%) | 62 (50.4%) |  |
| **Child-Pugh class** | A | | | | 91 (55.5%) | 82 (68.3%) | 0.074 |
|  | B | | | | 47 (28.7%) | 22 (18.3%) |  |
|  | C | | | | 26 (15.9%) | 16 (13.3%) |  |
| **Presence of complications of cirrhosis** | compensated | | | | 102 (60.4%) | 87 (70.7%) | 0.067 |
|  | decompensated | | | | 67 (39.6%) | 36 (29.3%) |  |
| **Charlson Comorbidity group** | CCI=0 | | | | 48 (28.4%) | 47 (38.2%) | 0.26 |
|  | CCI=1 | | | | 61 (36.1%) | 34 (27.6%) |  |
|  | CCI=2 | | | | 28 (16.6%) | 22 (17.9%) |  |
|  | CCI=3+ | | | | 32 (18.9%) | 20 (16.3%) |  |
| **Diabetes** | no diabetes | | | | 91 (53.8%) | 74 (60.2%) | 0.28 |
|  | diabetes | | | | 78 (46.2%) | 49 (39.8%) |  |
| **SF-36 scores (mean, SD)** | |  | | |  |  |  |
| Physical functioning | | |  | | 61.8 (31.3) | 63.6 (27.0) | 0.60 |
| Role limitations due to physical health | | |  | | 45.4 (42.2) | 43.8 (41.2) | 0.75 |
| Bodily pain | | |  | | 63.9 (32.1) | 62.3 (33.1) | 0.68 |
| General health | | |  | | 38.4 (19.0) | 38.0 (19.7) | 0.86 |
| Energy and fatigue | | |  | | 47.3 (27.3) | 46.1 (25.5) | 0.71 |
| Social functioning | | |  | | 68.7 (32.8) | 70.0 (32.5) | 0.73 |
| Role limitations due to emotional health | | | |  | 81.7 (26.8) | 78.5 (28.5) | 0.32 |
| Emotional well being | | | |  | 71.6 (23.2) | 73.2 (22.4) | 0.57 |

**Additional file 1: Table S3**. Sociodemographic and clinical characteristics of Indigenous Australians according to recruitment method: ‘second opportunity’ pathway (n=6) versus mainstream recruitment (all patients n=117 and Indigenous patients n=6)

|  |  | **Mainstream**  **Recruitment**  **(all patients)** | **Mainstream**  **Recruitment**  **(Indigenous patients)** | **‘Second opportunity’**  **pathway** | **Compared to**  **mainstream all patients** | **Compared to mainstream**  **Indigenous patients** |
| --- | --- | --- | --- | --- | --- | --- |
|  |  | N=117 | N=7 | N=6 | **p-value*** | **p-value*** |
| **Age (years)** mean (SD) |  | 60.8 (11.0) | 57.2 (15.1) | 57.9 (4.0 ) | 0.90 | 0.52 |
| **Gender** | Female | 40 (34.2%) | 3 (43%) | 2 (33%) | 1.00 | 1.00 |
|  | Male | 77 (65.8%) | 4 (57%) | 4 (67%) |  |  |
| **Education** | Junior High/less | 53 (45.3%) | 5 (71%) | 5 (83%) | 1.00 | 0.10 |
|  | Senior High/higher | 64 (54.7%) | 2 (29%) | 1 (17%) |  |  |
| **Socioeconomic status** | Q1 most affluent/Q2/Q3 | 68 (58.1%) | 3 (43%) | 1 (17%) | 0.56 | 0.09 |
|  | Q4/Q5Most disadvantage | 49 (41.9%) | 4 (57%) | 5 (83%) |  |  |
| **Remoteness of residence** | Major city area | 95 (81.2%) | 5 (71%) | 5 (83%) | 1.00 | 1.00 |
|  | Outside major city area | 22 (18.8%) | 2 (29%) | 1 (17%) |  |  |
| **Primary liver disease aetiology** | Alcohol | 43 (36.8%) | 4 (57%) | 1 (17%) | 0.48 | 0.50 |
|  | HCV/HBV | 31 (26.5%) | 2 (29%) | 3 (50%) |  |  |
|  | NAFLD/NASH + other | 43 (36.8%) | 1 (14%) | 2 (33%) |  |  |
| **Presence of complications of cirrhosis** | compensated | 84 (71.8%) | 6 (86%) | 3 (50%) | 0.27 | 0.36 |
|  | decompensated | 33 (28.2%) | 1 (14%) | 3 (50%) |  |  |
| **Diabetes** | no diabetes | 71 (60.7%) | 5 (71%) | 3 (50%) | 0.43 | 0.68 |
|  | diabetes | 46 (39.3%) | 2 (29%) | 3 (50%) |  |  |

* Fisher's exact;

**Additional file 1: Table S4**. Mean SF-36 scores according to patient knowledge

|  | **Poor knowledge**  **N=51 (%)** | **Good knowledge**  **N=72 (%)** |  |
| --- | --- | --- | --- |
| **SF-36 domains** | **Mean (SD)** | **Mean (SD)** | **p-value*** |
| Physical functioning | 55.6 (26.3) | 69.3 (26.2) | **0.011** |
| Role limitations due to physical health | 40.0 (40.4) | 46.5 (41.9) | 0.182 |
| Bodily pain | 60.9 (34.9) | 63.4 (31.9) | 0.412 |
| General health | 38.7 (19.4) | 37.5 (20.1) | 0.849 |
| Energy and fatigue | 44.4 (26.5) | 47.3 (25.0) | 0.320 |
| Social functioning | 68.5 (35.0) | 71.1 (30.9) | 0.882 |
| Role limitations due to emotional health | 76.7 (29.5) | 79.8 (27.9) | 0.742 |
| Emotional well being | 72.9 (23.5) | 73.4 (21.8) | 0.945 |

* Multivariable logistic regression adjusted for education level, socioeconomic status, presence of complications of cirrhosis and duration of cirrhosis.

**Additional file 1: Table S5**. Adjusted incidence rate ratios and cost ratios according to knowledge score: sensitivity analyses carried using a cut-off of 57.1% on the 8-item score; and using a cut-off of ≥60.0% on the 11-item survey among 121 patients ‡

|  | **Sensitivity analysis 1**  **(8 items, cut-off 57.1%)** | | **Sensitivity analysis 2**  **(11 items, cut-off 60.0%)** | |
| --- | --- | --- | --- | --- |
|  | **IRR (95%CI)*** | **p-value** | **IRR (95%CI)*** | **p-value** |
| **Data source: Queensland Hospital Admitted Patient Data Collection** |  |  |  |  |
| All-cause admission | 0.23 (0.19-0.28) | **<0.001** | 0.34 (0.28-0.41) | **<0.001** |
| Cirrhosis admission | 0.62 (0.37-1.04) | 0.068 | 0.78 (0.52-1.19) | 0.257 |
| Planned one-day admission (cirrhosis admission) | 3.65 (1.35-9.88) | **0.011** | 10.9 (4.17-28.55) | **<0.001** |
| Admitted via the emergency department (any admission) | 0.52 (0.35-0.77) | **0.001** | 0.59 (0.41-0.84) | **0.004** |
| Admitted via the emergency department (cirrhosis admission) | 0.53 (0.30-0.93) | **0.027** | 0.57 (0.35-0.93) | **0.025** |
| **Data source: Emergency Data Collection** | **IRR (95%CI)*** | **p-value** | **IRR (95%CI)*** | **p-value** |
| Emergency presentation (any reason) | 0.63 (0.45-0.87) | **0.005** | 0.77 (0.58-1.03) | 0.077 |
| Cirrhosis-related emergency presentation | 0.35 (0.17-0.74) | **0.006** | 0.47 (0.25-0.88) | **0.018** |
| **Data source: National Hospital Cost Data Collection** | **Cost ratio (95%CI)**** | **p-value** | **Cost ratio (95%CI)**** | **p-value** |
| Total cost for any admission | 0.08 (0.08-0.08) | **<0.001** | 0.19 (0.19-0.19) | **<0.001** |
| Total cost for cirrhosis admissions | 0.33 (0.32-0.33) | **<0.001** | 0.41 (0.40-0.41) | **<0.001** |

Note: Incidence rate ratio (IRR) and cost ratio with poor knowledge as reference group; ‡ Two patients for whom we did not have hospital admission data were excluded from these analyses; * Multivariable Poisson regression model included education level, socioeconomic status, presence of complications of cirrhosis and duration of cirrhosis; ** Multivariable Poisson regression model included education level, Charlson Comorbidity Index, and presence of complications of cirrhosis.
